# Improving the Rank Precision of Population Health Measures for Small Areas with Longitudinal and Joint Outcome Models

**DOI:** 10.1371/journal.pone.0130027

**Published:** 2015-06-22

**Authors:** Jessica K. Athens, Patrick L. Remington, Ronald E. Gangnon

**Affiliations:** 1 Department of Population Health Sciences, New York University School of Medicine, New York, New York, United States of America; 2 University of Wisconsin Population Health Institute, Madison, Wisconsin, United States of America; 3 Department of Population Health Sciences, University of Wisconsin, Madison, Wisconsin, United States of America; 4 Department of Biostatistics and Medical Informatics, University of Wisconsin, Madison, Wisconsin, United States of America; Medical University of Vienna, AUSTRIA

## Abstract

**Objectives:**

The University of Wisconsin Population Health Institute has published the *County Health Rankings* since 2010. These rankings use population-based data to highlight health outcomes and the multiple determinants of these outcomes and to encourage in-depth health assessment for all United States counties. A significant methodological limitation, however, is the uncertainty of rank estimates, particularly for small counties. To address this challenge, we explore the use of longitudinal and pooled outcome data in hierarchical Bayesian models to generate county ranks with greater precision.

**Methods:**

In our models we used pooled outcome data for three measure groups: (1) Poor physical and poor mental health days; (2) percent of births with low birth weight and fair or poor health prevalence; and (3) age-specific mortality rates for nine age groups. We used the fixed and random effects components of these models to generate posterior samples of rates for each measure. We also used time-series data in longitudinal random effects models for age-specific mortality. Based on the posterior samples from these models, we estimate ranks and rank quartiles for each measure, as well as the probability of a county ranking in its assigned quartile. Rank quartile probabilities for univariate, joint outcome, and/or longitudinal models were compared to assess improvements in rank precision.

**Results:**

The joint outcome model for poor physical and poor mental health days resulted in improved rank precision, as did the longitudinal model for age-specific mortality rates. Rank precision for low birth weight births and fair/poor health prevalence based on the univariate and joint outcome models were equivalent.

**Conclusion:**

Incorporating longitudinal or pooled outcome data may improve rank certainty, depending on characteristics of the measures selected. For measures with different determinants, joint modeling neither improved nor degraded rank precision. This approach suggests a simple way to use existing information to improve the precision of small-area measures of population health.

## Introduction

The *County Health Rankings*, first published in 2010 by the University of Wisconsin Population Health Institute, provide population health measures for nearly all United States counties. The *Rankings* are designed to direct media and policy-maker attention toward the multiple determinants of health and encourage in-depth community health assessment [1]. The community engagement and discussion about health and its determinants is intended to motivate the implementation of evidence-based programs to improve population health.

One challenge to the *Rankings* is its reliance on small-area estimates, which are commonly affected by small sample sizes, large standard errors, and statistical outliers. These features of small-area estimates lead to uncertainty regarding the health of counties—especially for small counties—in the *Rankings*. Hierarchical Bayesian models, however, can be used to improve small-area estimates of health-related measures and the resulting ranks. The benefits of Bayesian estimates are well-known: they draw in extreme values that are often statistical artifacts due to data sparsity by using information from related units [2]. Furthermore, Bayesian estimates allow us to estimate more adequately uncertainty in performance across units—a critical feature when comparing (or ranking) entities [3].

Bayesian estimates can be generated from hierarchical models with no fixed effects (empty models), or estimates can be informed by adding covariates to the model which, in most cases, improve their precision [4]. Longitudinal and joint outcome models are another way to leverage information and estimate more accurately county-level health measures and ranks. Longitudinal data are ideally suited for hierarchical models, as data from different time points are nested within counties. Joint outcome or shared component models are a generalization of the longitudinal model. Two or more measures that share common determinants (i.e., risk factors) can be modeled together in order to pool data and “borrow strength” for more reliable point and rank estimation [5,6]. These features of joint or longitudinal modeling could prove valuable for the *Rankings*, as earlier work has demonstrated that applying univariate hierarchical Bayesian models result in limited improvement for rank precision relative to ranks based on observed data, particularly for middle-ranked counties [4].

## Methods

### Data

*The County Health Rankings* report data for more than 25 health-related measures. Five of these measures represent the “health outcomes” or the current health of a community. Health outcomes measures include premature mortality, self-reported fair or poor health prevalence, average poor mental health days per month, average poor physical health days per month, and percent of live births with low birth weight. The remaining measures in the *Rankings* model represent the determinants of these health outcomes and are divided into four categories: health care, health behaviors, socioeconomic factors, and the physical environment [7]. Not all of the measures have longitudinal data available or are appropriate for modeling together, so we restricted our analyses to the five health outcome measures. More information on the measures and methods used in the *County Health Rankings* is available at www.countyhealthrankings.org.

The measures and data sources for health outcomes in the 2010 *Rankings* are listed in Table 1. For all measures but premature mortality, the analyses used National Vital Statistics System (NVSS) and Behavioral Risk Factor Surveillance System (BRFSS) data aggregated to the county level and reported to the Institute.

**Table 1:** Data Used in Longitudinal and Joint Outcome Models.

Measure	Source	Years
Mortality and population counts by age group	Underlying cause of mortality query, CDC Wide-ranging Online Data for Epidemiologic Research (WONDER)	1995–1997; 1998–2000; 2001–2003; 2004–2006*
Mean poor physical health days per month	Behavioral Risk Factor Surveillance System (BRFSS)	2002–2008
Mean poor mental health days per month	BRFSS	2002–2008
Percent reporting fair or poor health	BRFSS	2002–2008
Percent of live births with low birth weight births	National Vital Statistics System, National Center for Health Statistics	2000–2006

* 2004–2006 data were used in joint outcome models of age-specific mortality.

The *Rankings* represent premature mortality by the rate of years of potential life lost before the age of 75 (YPLL-75) per 100,000 population, where each death in a county is weighted based on the difference between age 75 and the age at death [8]. Because YPLL-75 rates are a composite of age-specific death rates, we directly modeled the underlying mortality rates for nine age groups and used the resulting output to calculate posterior samples of YPLL-75. Raw mortality and population counts for age < 1, ages 1–4, and 10-year age groups between the ages of 5 to 74, were accessed from the CDC WONDER underlying cause of death query system for four 3-year time periods: 1995–97, 1998–2000, 2001–03, and 2004–06 [9].

The percentage of counties with missing health outcome data ranged from 3.1% to 13.7%. Counties with missing data were disproportionately rural. Values for vital statistics measures—YPLL-75 and low birth weight births—were suppressed if based on five or fewer events. BRFSS censored values for counties with fewer than 50 respondents or a 95% confidence interval width greater than 20% of the point estimate.

### Longitudinal model

Mortality data for each of nine age groups included number of events (y) and population denominator (n) for the years 1995–97 (t = -3), 1998–2000 (t = -2), 2001–03 (t = -1), and 2004–06 (t = 0). Estimates from the years 2004–06 were our primary interest, so we set that time period as our intercept. For each age group, we fit the following generalized linear mixed effects log-linear regression models for age-specific mortality with state- and county-level random intercepts and slopes.

$$y_{jkt}\sim Poisson(\rho_{jkt},n_{jkt})$$

$$log\left(\rho_{jkt}\right)=\alpha +a_{k}+a_{jk}+(\beta +b_{k}+b_{jk})\times t$$

$$a_{k}\sim N(0,\sigma_{k}^{2})$$

$$a_{jk}\sim N(0,\sigma_{jk}^{2})$$

$$b_{k}\sim N(0,\tau_{k}^{2})$$

$$b_{jk}\sim N(0,\tau_{jk}^{2})$$

Where

*y*_*jkt*_ = number of deaths in county *j* in state *k* during year *t*

*n*_*jkt*_ = population for age group in county *j* in state *k* during year *t*

*ρ*_*jkt*_ = mortality rate for age group in county *j* in state *k* during year *t*

*α* = fixed effects intercept parameter for mortality at time = 0 (2004–06)

*a*_*k*_ = state-specific random intercept parameter

$\sigma_{k}^{2}$ = variance of state-specific random intercept parameters

*a*_*jk*_ = county-specific random intercept parameter

$\sigma_{jk}^{2}$ = variance of county-specific random intercept parameters

*β* = fixed effects slope parameter for time

*b*_*k*_ = state-specific random slope parameter

$\tau_{k}^{2}$ = variance of state-specific random slope parameters

*b*_*jk*_ = county-specific random slope parameter

$\tau_{jk}^{2}$ = variance of county-specific random slope parameters

for *j* = 1, 2, …, *m*_*k*_; *k* = 1, 2, …, 51; and *t* = -3 (1995–97), -2 (1998–2000), -1 (2001–03), 0 (2004–06)

We used the resulting posterior samples of age-specific death rates in 2004–06 (*ρ*_*jkt*_) to calculate premature mortality (YPLL-75) rates by county. For more information on the calculation and use of YPLL rates, see Wise et al., 1988 [8] and Gardner and Sanborn, 1990 [10].

### Joint outcome models

We also examined the performance of joint outcome models for 2004–06 age-specific mortality data—borrowing strength across age groups rather than over time—and for two additional sets of measures: (1) average poor physical and poor mental health days per month and (2) percent reporting fair or poor health and percent of live births with low birth weight. In our measure dyads (1) and (2) we chose to jointly model measures that followed the same distribution (e.g., Gaussian, binomial). Although one could jointly model measures from different families of distributions [5,11], we do not consider such models in this paper.

The first measure pair was selected due to the positive correlation between average poor mental health days and poor physical health days per month. High county-level averages for poor mental and poor physical health days are both strongly associated with low levels of physical activity, high poverty rates, and inadequate social support [12]. Allostatic load or stress response is considered a common determinant for mental and physical health [13]. Though—unlike the model for mortality rates—this joint modeling does not fundamentally increase sample size, we hypothesized that including more information per respondent in a joint outcome model could improve the accuracy of the resulting estimates for each measure. The second measure pair—fair/poor health prevalence and low birth weight births—was chosen to demonstrate that, for unrelated or weakly related measures, the joint modeling procedure will neither improve nor degrade the accuracy of the resulting estimates.

#### Premature mortality

Using cross-sectional mortality data (2004–06), we created the following joint outcome model for premature mortality across the 9 age groups.

$$y_{ajk}\sim Poisson(\rho_{ajk},n_{ajk})$$

$$log(\rho_{ajk})=\alpha +a_{k}+a_{jk}+(\beta_{a}+b_{ak}+b_{ajk})\times a$$

$$a_{k}\sim N(0,\sigma_{k}^{2})$$

$$a_{jk}\sim N(0,\sigma_{jk}^{2})$$

$$b_{ak}\sim N(0,\tau_{ak}^{2})$$

$$b_{ajk}\sim N(0,\tau_{ajk}^{2})$$

Where

*y*_*ajk*_ = number of deaths for age group *a* in county *j* in state *k*

*n*_*ajk*_ = population for age group *a* in county *j* in state *k*

*ρ*_*ajk*_ = mortality rate for age group *a* in county *j* in state *k*

*α* = fixed effects intercept parameter for reference age group (age <1 year)

*a*_*k*_ = state-specific random intercept parameter for reference age group

$\sigma_{k}^{2}$ = variance of state-specific random intercept parameters for reference age group

*a*_*jk*_= county-specific random intercept parameter for reference age group

$\sigma_{jk}^{2}$ = variance of county-specific random intercept parameters for reference age group

*β*_*a*_ = fixed effects slope parameter for age group *a*

*b*_*ak*_ = state-specific random slope parameter for age group *a*

$\tau_{ak}^{2}$ = variance of state-specific random slope parameters for age group *a*

*b*_*ajk*_ = county-specific random slope parameter for age group *a*

$\tau_{ajk}^{2}$ = variance of county-specific random slope parameters for age group *a*

for *a* = 1, 2, …, 9; *j* = 1, 2, …, *m*_*k*_; *k* = 1, 2, …, 51

#### Average poor physical and poor mental health days

Average number of poor physical health days per month and the average number of poor mental health days per month were reported from the BRFSS. Data reported included the (observed) mean value by county (*m*), the number of respondents (*n*), and the 95% confidence limits for the county-specific means, from which standard errors (*s*) could be calculated. Based on the Central Limit Theorem, the sampling distribution of the observed county-specific mean value is approximately normal regardless of the underlying distribution of poor physical and poor mental health days. Using these data, we fit the following joint model for these outcomes.

$$\log({\text{m}}_{\text{ijk}})\sim \text{Normal}(\mu_{\text{ijk}},s_{ijk}^{2})$$

$$\log(\mu_{\text{ijk}})=\alpha +{\text{a}}_{\text{k}}+{\text{a}}_{\text{jk}}+(\beta_{\text{i}}+{\text{b}}_{\text{k}}+{\text{b}}_{\text{jk}})\times \text{i}$$

$$a_{k}\sim N(0,\sigma_{k}^{2})$$

$$a_{jk}\sim N(0,\sigma_{jk}^{2})$$

$$b_{k}\sim N(0,\tau_{k}^{2})$$

$$b_{jk}\sim N(0,\tau_{jk}^{2})$$

Where

m_ijk_ = observed mean for outcome *i* in county *j* in state *k*

μ_ijk_ = population mean for outcome *i* in county *j* in state *k*

$s_{ijk}^{2}$ = sampling variance for log(m_ijk_)

*α* = fixed effects intercept parameter for reference measure (poor physical health days)

a_k_ = state-specific random intercept parameter for reference measure

$\sigma_{k}^{2}$ = variance of state-specific random intercept parameters for reference measure

a_jk_ = county-specific random intercept parameter for reference measure

$\sigma_{jk}^{2}$ = variance of county-specific random intercept parameters for reference measure

β_i_ = fixed effects slope parameter for poor mental health days

b_k_ = state-specific random slope parameter for poor mental health days

$\tau_{k}^{2}$ = variance of state-specific random slope parameters for poor mental health days

b_jk_ = county-specific random slope parameter for poor mental health days

$\tau_{jk}^{2}$ = variance of county-specific random slope parameters for poor mental health days

for *i* = 0, 1; *j* = 1, 2, …, *m*_*k*_; *k* = 1, 2, …, 51

#### Fair or poor health prevalence and percent low birth weight births

Data on fair or poor health prevalence, reported from BRFSS, consisted of a point estimate (*p*_0*jk*_) with nominal 95% confidence limits, which were used to calculate the county-specific standard error of *p*_0*jk*_ (*s*_0*jk*_). From the estimated standard error, we determined the effective sample size: $n_{0jk}=p_{0jk}(1-p_{0jk})/s_{0jk}^{2}$. Data on low birth weight births consisted of a census of all live births (*n*_1*jk*_), a count of births for which birth weight was less than 2500 grams (*y*_1*jk*_) and the observed proportion of low birth weight births (*p*_1*jk*_ = *y*_1*jk*_/*n*_1*jk*_). The following joint logistic regression model was fit for these outcomes.

$${\text{y}}_{\text{ijk}}\sim \text{binomial}(\pi_{\text{ijk}},{\text{n}}_{\text{ijk}})$$

$${\text{y}}_{\text{ijk}}=\alpha +{\text{a}}_{k}+{\text{a}}_{jk}+(\beta_{\text{i}}+{\text{b}}_{\text{k}}+{\text{b}}_{\text{jk}})\times \text{i}$$

$$a_{k}\sim N(0,\sigma_{k}^{2})$$

$$a_{jk}\sim N(0,\sigma_{jk}^{2})$$

$$b_{k}\sim N(0,\tau_{k}^{2})$$

$$b_{jk}\sim N(0,\tau_{jk}^{2})$$

Where

y_ijk_ = count of outcome *i* in county *j* in state *k*

π_ijk_ = population proportion for outcome *i* in county *j* in state *k*

n_ijk_ = (effective) sample size for outcome *i* in county *j* in state *k*

*α* = fixed effects intercept parameter for reference measure (fair or poor health prevalence)

a_*k*_ = state-specific random intercept parameter for reference measure

$\sigma_{k}^{2}$ = variance of state-specific random intercept parameters for reference measure

a_*jk*_ = county-specific random intercept parameter for reference measure

$\sigma_{jk}^{2}$ = variance of county-specific random intercept parameters for reference measure

β_i_ = fixed effects slope parameter for percent low birth weight births

*b*_*k*_ = state-specific random slope parameter for low birth weight births

$\tau_{k}^{2}$ = variance of state-specific random slope parameters for low birth weight births

*b*_*jk*_ = county-specific random slope parameter for low birth weight births

$\tau_{jk}^{2}$ = variance of county-specific random slope parameters for low birth weight births

for *i* = 0, 1; *j* = 1, 2, …, m_*k*_; *k* = 1, 2, …, 51

### Cross-sectional models

In addition to the longitudinal and joint outcome models described above, each of the five health outcomes measures were estimated using cross-sectional, univariate data in a generalized linear mixed effects model with an intercept as fixed effect, and state- and county-level random effects. An example of the Poisson model specification is below.

$$y_{jk}\sim Poisson(\rho_{jk},n_{jk})$$

$$log(\rho_{jk})=\beta_{0}+e_{k}+e_{jk}$$

$$e_{k}\sim N(0,\sigma_{k}^{2})$$

$$e_{jk}\sim N(0,\sigma_{jk}^{2})$$

Where

*y*_*jk*_ = number of events in county *j* within state *k*

*n*_*jk*_ = population county *j* within state *k*

*ρ*_*jk*_ = event rate for county *j* in state *k*

*β*_0_ = Intercept, or the average log event rate across all counties in all states

*e*_*k*_ = state-specific random effect parameter

$\sigma_{k}^{2}$ = variance of state-specific random effects

*e*_*jk*_ = county-specific random effect parameter

$\sigma_{jk}^{2}$ = variance of county-specific random effects

For *j* = 1, 2, …, *m*_*k*_; *k* = 1, 2, …, 51

### Estimation and model fit

Model parameters (regression coefficients and variances of the state- and county-level random effects) were estimated by maximum likelihood. Empirical Bayes estimates of the random effects were obtained by conditioning on the estimated variance parameters. Samples from the joint posterior distribution of the regression coefficients, state-level random effects, and county-level random effects were drawn from (multivariate) normal approximations to their conditionally independent posterior distributions. Posterior samples of the county-specific measures were then derived from these samples. Estimation and simulations were performed in R (version 2.15.1), using the lme4 and MASS libraries [14–16].

To test our models’ performance, we used random number generation procedures for binomial, Poisson, and normal distributions to produce posterior predictive data sets based on the posterior samples and the reported population data for each measure. Similarities between parameters calculated from the posterior predictive data sets and the original data were quantified by posterior predictive p-values. These p-values are similar to those in frequentist statistics, in which a probability is set as a threshold below which the null hypothesis is rejected. In this case, a significant p-value (p < 0.05) suggested a poor fit [17,18]. Mean values would be replicated even with a poorly fitted model, so we selected inter-quartile range and skew as summary measures of a variable’s distribution.

### Ranking

Posterior samples of county-specific ranks in the entire nation were obtained by ranking each draw of the county-specific means or rates. Point estimates of ranks were obtained by ranking the posterior mean ranks, which is equivalent to minimizing squared error loss on the ranks. (See Louis, 2001 for the comparative advantages of different loss functions using ranks [19].) We used these ranks to assign counties to (national) quartiles of performance: quartile 1 represents the healthiest counties, quartile 4 the least healthy. The probability of a county ranking in its assigned quartile across the posterior samples was also calculated as a means to represent rank certainty. We then mapped the posterior probability of a county ranking in its assigned quartile using the maps library in R (2.15.1) [20].

## Results

### Estimates and model fit

For premature deaths, our posterior predictive checks initially suggested that the joint outcome model—modeling mortality rates for all age groups simultaneously—best captured the distribution of the original data. The posterior predictive p-values for inter-quartile range and skew were both insignificant (p > 0.05), suggesting good model fit. The cross-sectional and longitudinal models, conversely, were unable to capture the dispersion of observed premature mortality rates, represented by highly significant posterior predictive p-values for inter-quartile range. However, posterior predictive p-values for individual age-group mortality rates indicate that the joint outcome model performed poorly at replicating the original data for the following age groups: ages 1–4, ages 5–14, and ages 65–74. In contrast, posterior predictive p-values for individual age-group mortality rates based on the cross-sectional and longitudinal models were all > 0.05 and indicative of reasonable model fit.

In our two other joint outcomes models, we saw some improvement in model fit for poor physical health days using the joint outcome model. The posterior predictive checks indicated that samples from the joint outcomes model better captured the dispersion in the observed data for poor physical health days, but there was little to no improvement in model fit for poor mental health days. For the final measure pair, fair/poor health prevalence and percent of births with low birth weight, the model fits were equivalent between the cross-sectional and joint outcome models. For fair/poor health prevalence, both models successfully replicated the interquartile range and skew of the original data; for low birth weight births, the models captured the dispersion of the observed measure, but not the skew. (See Tables 2–9 for estimates of fixed and random effects from all measure-model combinations.)

**Table 2:** Univariate Model Fits for Infant, Ages 1–4, and Ages 5–14 Mortality Rates.

	Cross-sectional Models			Longitudinal Models			
**Infant mortality (age < 1 year): Random effects**							
Groups	Parameter	Var.	SD	Parameter	Var.	SD	Corr.
*County*	*Intercept*	0.046	0.215	*Intercept*	0.044	0.210	
*Year*	—	—	—		0.0017	0.041	0.355
*State*	*Intercept*	0.039	0.197	*Intercept*	0.038	0.196	
*Year*	—	—	—		0.0004	0.021	0.728
**Infant mortality (age < 1 year): Fixed effects**							
	Est.	SE	p-value	Est.	SE	p-value	
*Intercept*	-5.00	0.029	<0.0001	-5.01	0.029	<0.0001	
*Year*	—	—	—	-0.02	0.004	<0.0001	
				*Corr (fixed effects)*		0.63	
**Ages 1–4 mortality: Random effects**							
Groups	Parameter	Var.	SD	Parameter	Var.	SD	Corr.
*County*	*Intercept*	0.048	0.220	*Intercept*	0.048	0.219	
*Year*	—	—	—		0.0016	0.040	0.162
*State*	*Intercept*	0.063	0.250	*Intercept*	0.063	0.251	
*Year*	—	—	—		0.0005	0.022	0.949
**Ages 1–4 mortality: Fixed effects**							
	Est.	SE	p-value	Est.	SE	p-value	
*Intercept*	-8.10	0.039	<0.0001	-8.10	0.037	<0.0001	
*Year*	—	—	—	-0.08	0.005	<0.0001	
				*Corr (fixed effects)*		0.69	
**Ages 5–14 mortality: Random effects**							
Groups	Parameter	Var.	SD	Parameter	Var.	SD	Corr.
*County*	*Intercept*	0.062	0.249	*Intercept*	0.054	0.233	
*Year*	—	—	—		0.0012	0.035	0.307
*State*	*Intercept*	0.047	0.217	*Intercept*	0.052	0.229	
*Year*	—	—	—		0.0003	0.017	0.610
**Ages 5–14 mortality: Fixed effects**							
	Est.	SE	p-value	Est.	SE	p-value	
*Intercept*	-8.66	0.034	<0.0001	-8.64	0.034	<0.0001	
*Year*	—	—	—	-0.09	0.004	<0.0001	
				*Corr (fixed effects)*		0.47	

**Table 3:** Univariate Model Fits for Ages 15–24, 25–34, and Ages 35–34 Mortality Rates.

	Cross-sectional Models			Longitudinal Models			
**Ages 15–24 mortality: Random effects**							
Groups	Parameter	Var.	SD	Parameter	Var.	SD	Corr.
*County*	*Intercept*	0.103	0.320	*Intercept*	0.096	0.309	
*Year*	—	—	—		0.0022	0.047	0.170
*State*	*Intercept*	0.049	0.222	*Intercept*	0.050	0.224	
*Year*	—	—	—		0.0009	0.031	0.380
**Ages 15–24 mortality: Fixed effects**							
	Est.	SE	p-value	Est.	SE	p-value	
*Intercept*	-7.03	0.033	<0.0001	-7.00	0.033	<0.0001	
*Year*	—	—	—	-0.01	0.005	0.009	
				*Corr (fixed effects)*		0.37	
**Ages 25–34 mortality: Random effects**							
Groups	Parameter	Var.	SD	Parameter	Var.	SD	Corr.
*County*	*Intercept*	0.075	0.274	*Intercept*	0.074	0.272	
*Year*	—	—	—		0.0033	0.057	0.355
*State*	*Intercept*	0.062	0.248	*Intercept*	0.058	0.242	
*Year*	—	—	—		0.0010	0.032	0.546
**Ages 25–34 mortality: Fixed effects**							
	Est.	SE	p-value	Est.	SE	p-value	
*Intercept*	-6.80	0.036	<0.0001	-6.81	0.035	<0.0001	
*Year*	—	—	—	-0.02	0.005	<0.0001	
				*Corr (fixed effects)*		0.52	
**Ages 35–44 mortality: Random effects**							
Groups	Parameter	Var.	SD	Parameter	Var.	SD	Corr.
*County*	*Intercept*	0.070	0.264	*Intercept*	0.070	0.264	
*Year*	—	—	—		0.0031	0.056	0.421
*State*	*Intercept*	0.054	0.231	*Intercept*	0.055	0.235	
*Year*	—	—	—		0.0012	0.034	0.380
**Ages 35–44 mortality: Fixed effects**							
	Est.	SE	p-value	Est.	SE	p-value	
*Intercept*	-6.19	0.034	<2e-16	-6.18	0.034	<0.0001	
*Year*	—	—	—	0.02	0.005	0.003	
				*Corr (fixed effects)*		0.38	

**Table 4:** Univariate Model Fits for Ages 45–54, 55–64, and Ages 65–74 Mortality Rates.

	Cross-sectional Models			Longitudinal Models			
**Ages 45–54 mortality: Random effects**							
Groups	Parameter	Var.	SD	Parameter	Var.	SD	Corr.
*County*	*Intercept*	0.048	0.220	*Intercept*	0.049	0.220	
*Year*	—	—	—		0.0011	0.034	0.491
*State*	*Intercept*	0.048	0.219	*Intercept*	0.048	0.219	
*Year*	—	—	—		0.0004	0.021	0.626
**Ages 45–54 mortality: Fixed effects**							
	Est.	SE	p-value	Est.	SE	p-value	
*Intercept*	-5.45	0.032	<0.0001	-5.45	0.031	<0.0001	
*Year*	—	—	—	0.02	0.003	<0.0001	
				*Corr (fixed effects)*		0.59	
**Ages 55–64 mortality: Random effects**							
Groups	Parameter	Var.	SD	Parameter	Var.	SD	Corr.
*County*	*Intercept*	0.037	0.193	*Intercept*	0.037	0.192	
*Year*	—	—	—		0.0009	0.031	0.655
*State*	*Intercept*	0.032	0.178	*Intercept*	0.031	0.177	
*Year*	—	—	—		0.0003	0.018	0.719
**Ages 55–64 mortality: Fixed effects**							
	Est.	SE	p-value	Est.	SE	p-value	
*Intercept*	-4.69	0.026	<0.0001	-4.69	0.026	<0.0001	
*Year*	—	—	—	-0.04	0.003	<0.0001	
				*Corr (fixed effects)*		0.69	
**Age 65–74 Mortality Random effects**							
Groups	Parameter	Var.	SD	Parameter	Var.	SD	Corr.
*County*	*Intercept*	0.019	0.140	*Intercept*	0.019	0.139	
*Year*	—	—	—		0.0006	0.024	0.574
*State*	*Intercept*	0.016	0.127	*Intercept*	0.016	0.128	
*Year*	—	—	—		0.0002	0.014	0.634
**Ages 65–74 mortality: Fixed effects**							
	Est.	SE	p-value	Est.	SE	p-value	
*Intercept*	-3.83	0.018	<2e-16	-3.82	0.018	<0.0001	
*Year*	—	—	—	-0.05	0.002	<0.0001	
				*Corr (fixed effects)*		0.61	

**Table 5:** Joint Outcome Model Fit for Age-specific Mortality Rates.

Random effects	Parameter		Var.	SD	Fixed effects	Est.	SE	p-value	
Groups									
*County*	*Infants (Intercept)*		0.031	0.177	*Infants (Intercept)*	-4.94	0.028	<0.0001	
**	*Age 1–4 (m1)*		0.009	0.095	*Age 1–4 (m1)*	-3.15	0.025	<0.0001	
**	*Age 5–14 (m2)*		0.020	0.140	*Age 5–14 (m2)*	-3.71	0.024	<0.0001	
**	*Age 15–24 (m3)*		0.061	0.248	*Age 15–24 (m3)*	-2.09	0.021	<0.0001	
**	*Age 25–34 (m4)*		0.025	0.157	*Age 25–34 (m4)*	-1.86	0.021	<0.0001	
**	*Age 35–44 (m5)*		0.014	0.118	*Age 35–44 (m5)*	-1.25	0.016	<0.0001	
**	*Age 45–54 (m6)*		0.006	0.080	*Age 45–54 (m6)*	-0.50	0.013	<0.0001	
**	*Age 55–64 (m7)*		0.003	0.057	*Age 55–64 (m7)*	0.26	0.012	<0.0001	
**	*Age 65–74 (m8)*		0.007	0.086	*Age 65–74 (m8)*	1.11	0.015	<0.0001	
*State*	*Infants (Intercept)*		0.038	0.195					
	*Age 1–4 (m1)*		0.023	0.152					
	*Age 5–14 (m2)*		0.021	0.146					
	*Age 15–24 (m3)*		0.018	0.132					
	*Age 25–34 (m4)*		0.018	0.136					
	*Age 35–44 (m5)*		0.010	0.100					
	*Age 45–54 (m6)*		0.007	0.085					
	*Age 55–64 (m7)*		0.006	0.075					
	*Age 65–74 (m8)*		0.010	0.102					
Corr. (fixed effects)									
	*(Intr)*	*m1*	*m2*	*m3*	*m4*	*m5*	*m6*		*m7*
*m1*	-0.026								
*m2*	-0.028	0.028							
*m3*	-0.036	0.033	0.036						
*m4*	-0.036	0.034	0.037	0.045					
*m5*	-0.049	0.046	0.050	0.061	0.062				
*m6*	-0.058	0.055	0.060	0.073	0.074	0.100			
*m7*	-0.066	0.062	0.067	0.082	0.084	0.113	0.135		
*m8*	-0.052	0.048	0.052	0.064	0.065	0.088	0.105		0.119

**Table 6:** Univariate Model Fits for Fair or Poor Health Prevalence and Percent of Births with Low Birth Weight.

**Random effects**	**Fair or Poor Health Prevalence**			**Percent of Births with Low Birth Weight**		
Groups	Parameter	Var.	SD	Parameter	Var.	SD
*County*	*Intercept*	0.069	0.262	*Intercept*	0.021	0.144
*State*	*Intercept*	0.066	0.257	*Intercept*	0.034	0.184
**Fixed effects**	**Fair or Poor Health Prevalence**			**Percent of Births with Low Birth Weight**		
	Est.	SE	p-value	Est.	SE	p-value
*Intercept*	-1.70	0.037	<0.0001	-2.50	0.026	<0.0001

**Table 7:** Joint Outcome Model Fit for Fair or Poor Health Prevalence and Percent of Births with Low Birth Weight.

**Random effects**				
Groups	Parameter	Var.	SD	Corr
*County*	*Intercept* ^a^	0.069	0.262	
	*measure* ^*b*^	0.062	0.250	-0.844
*State*	*Intercept*	0.066	0.257	
	*measure*	0.038	0.194	-0.703
**Fixed effects**				
	Estimate	SE	p-value	
*Intercept*	-1.70	0.037	<0.0001	
*Measure*	-0.80	0.038	<0.0001	
	*Corr*. *(fixed effects)*		-0.71	

^a^ Fair or poor health prevalence is represented by the intercept (measure = 0).

^b^ Low birth weight is represented by measure = 1. Its national average across counties is estimated as the sum of the intercept and slope for measure.

**Table 8:** Univariate Model Fits for Average Poor Mental Health Days and Average Poor Physical Health Days.

**Random effects**	**Average Poor Mental Health Days**			**Average Poor Physical Health Days**		
Groups	Parameter	Var.	SD	Parameter	Var.	SD
*County*	*Intercept*	0.052	0.229	*Intercept*	0.048	0.218
*State*	*Intercept*	0.020	0.142	*Intercept*	0.021	0.144
**Fixed effects**	**Average Poor Mental Health Days**			**Average Poor Physical Health Days**		
	Est.	SE	p-value	Est.	SE	p-value
*Intercept*	1.17	0.021	<0.0001	1.24	0.021	<0.0001

**Table 9:** Joint Outcome Model Fit for Average Poor Mental Health Days and Average Poor Physical Health Days.

**Random effects**				
Groups	Parameter	Var.	SD	Corr
*County*	*Intercept* ^a^	0.038	0.196	
	*measure* ^*b*^	0.038	0.195	-0.313
*State*	*Intercept*	0.017	0.130	
	*measure*	0.002	0.049	0.086
**Fixed effects**				
	Estimate	SE	p-value	
*Intercept*	1.24	0.019	<0.0001	
*Measure*	-0.07	0.008	<0.0001	
	*Corr*. *(fixed effects)*		0.016	

^a^ Average poor physical health days are represented by the intercept (measure = 0).

^b^ Poor mental health days are represented by measure = 1. The national average across counties is estimated as the sum of the intercept and slope for measure.

### Rank estimates

We used the posterior ranks to determine the probability of a county ranking in a quartile as a means to explore rank precision. For each measure-model combination, we assigned counties to a quartile based on their mean percentile rank and calculated the probability with which each county ranked in its assigned quartile. This probability typically ranged between 0.30 and 1.0, and reflects the degree of certainty with which we know a county’s rank. Among counties within a quartile, we divided them into three classes based on the probability (certainty) with which they ranked in that quartile: low certainty (p < 0.50), medium certainty (p = 0.50–0.75), and high certainty (p ≥ 0.75).

Estimates of county ranks for premature death are similar across the three models considered (cross-sectional, longitudinal and joint outcomes). Based on the posterior probabilities of counties ranking in their assigned quartile, ranks are most precise using the longitudinal model and least precise using the joint outcomes model. Table 10 shows that the percent of counties ranking in their assigned quartiles with high probability (p ≥ 0.75) is highest for the longitudinal model (71.1% of all counties) and lowest for the joint outcomes model (46.8% of all counties) (Table 10). The improvement in precision with the longitudinal model over the cross-sectional model is expected. The degradation in performance of the joint outcomes model relative to the univariate model, though surprising, may reflect challenges in model fit for the age groups with low mortality rates.

**Table 10:** Percent and Number of Counties with High Probability (>75%) of Ranking in Assigned Quartile by Model and Measure.

Measure/Model		Q1	Q2	Q3	Q4	Total
Premature deaths: Independent	%	19.0	9.6	10.8	19.7	59.1
	N	597	301	337	620	1855
Premature deaths: Longitudinal	%	20.0	14.0	15.8	21.4	71.1
	N	627	439	496	671	2233
Premature deaths: Joint outcome	%	16.9	5.9	6.1	17.9	46.8
	N	532	185	191	562	1470
Poor physical health days: Independent	%	17.1	5.2	2.2	11.9	36.4
	N	536	163	68	375	1142
Poor physical health days: Joint outcome	%	17.5	5.7	2.3	13.5	38.9
	N	548	179	72	423	1222
Poor mental health days: Independent	%	17.8	8.1	3.8	14.7	44.4
	N	560	255	119	460	1394
Poor mental health days: Joint outcome	%	18.0	9.4	5.8	16.6	49.8
	N	566	295	182	522	1565
Fair/poor health prevalence: Independent	%	13.7	3.2	4.1	14.6	35.6
	N	430	101	130	457	1118
Fair/poor health prevalence: Joint outcome	%	14.0	3.7	4.7	14.9	37.4
	N	440	117	148	469	1174
Low birth weight births: Independent	%	14.4	4.4	6.6	18.2	43.5
	N	451	138	206	571	1366
Low birth weight births: Joint outcome	%	14.5	4.7	6.8	18.5	44.5
	N	455	147	215	581	1398

Joint modeling of poor physical and poor mental health days produced marginally better rank precision for poor mental health days compared with the univariate model. Using a joint outcomes approach, approximately 50% of counties ranked with high probability in their assigned quartile for poor mental health days, compared to 44% of counties with a univariate approach. However, improvements across counties for poor physical health days are marginal. Exploiting the association between these two measures improved our ability to differentiate the counties in terms of performance on poor mental health days and, to a lesser degree, on poor physical health days (Table 10).

As expected, joint modeling of fair or poor health prevalence and percent of births with low birth weight had little, if any, impact on the precision of the estimated ranks relative to separate univariate models for the two outcomes. The percent of counties ranking with high certainty in their assigned quartiles is the similar between the joint outcome and univariate models, indicating no improvement—and importantly no degradation—in rank precision.

### Maps of rank performance

**Fig 1:**
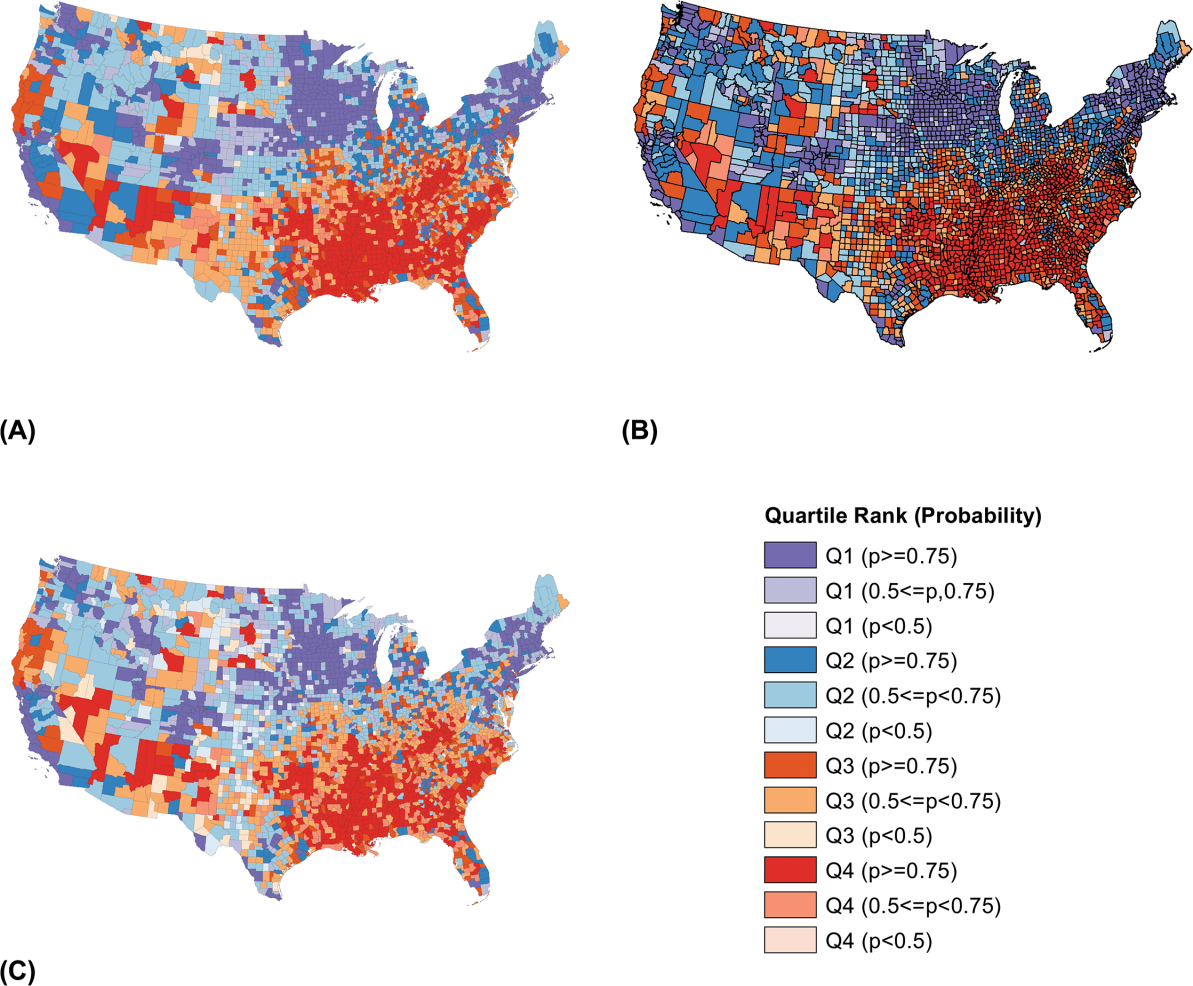
Choropleth Maps of U.S. County Rank Performance in Premature Deaths. Results based on independent model (A), longitudinal model (B), and joint outcome model (C).

In the following choropleth maps, we used four colors (purple, blue, orange, red) to represent the quartiles. The color ramps indicate precision; greater saturation indicates greater certainty that the county ranks in its assigned quartile. Maps of quartiles for all measure-model combinations are shown in Figs 1 through 4B.

**Fig 2:**
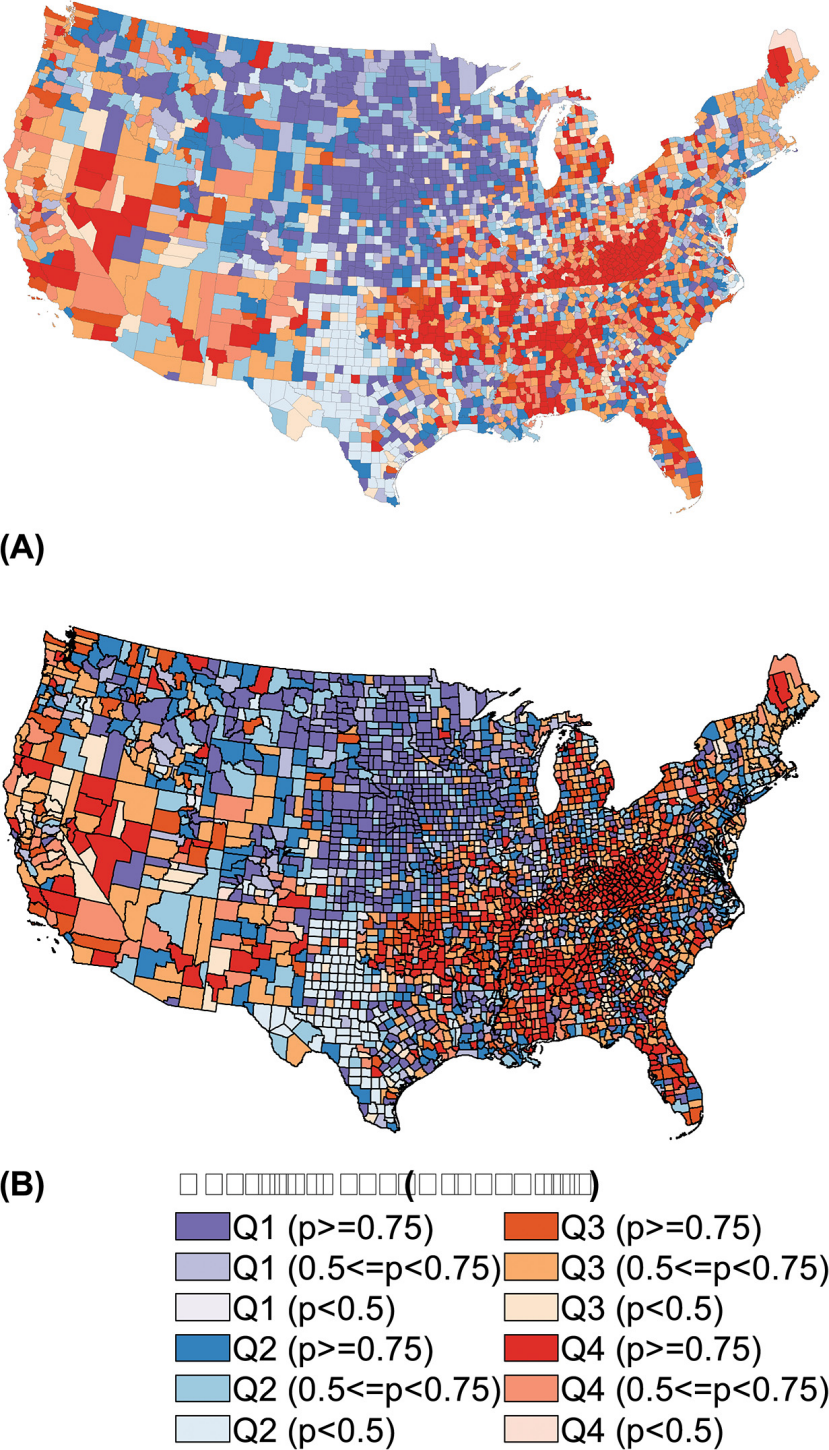
Choropleth Maps of U.S. County Rank Performance in Average Poor Mental Health Days. Results based on independent model (A) and joint outcome model (B).

**Fig 3:**
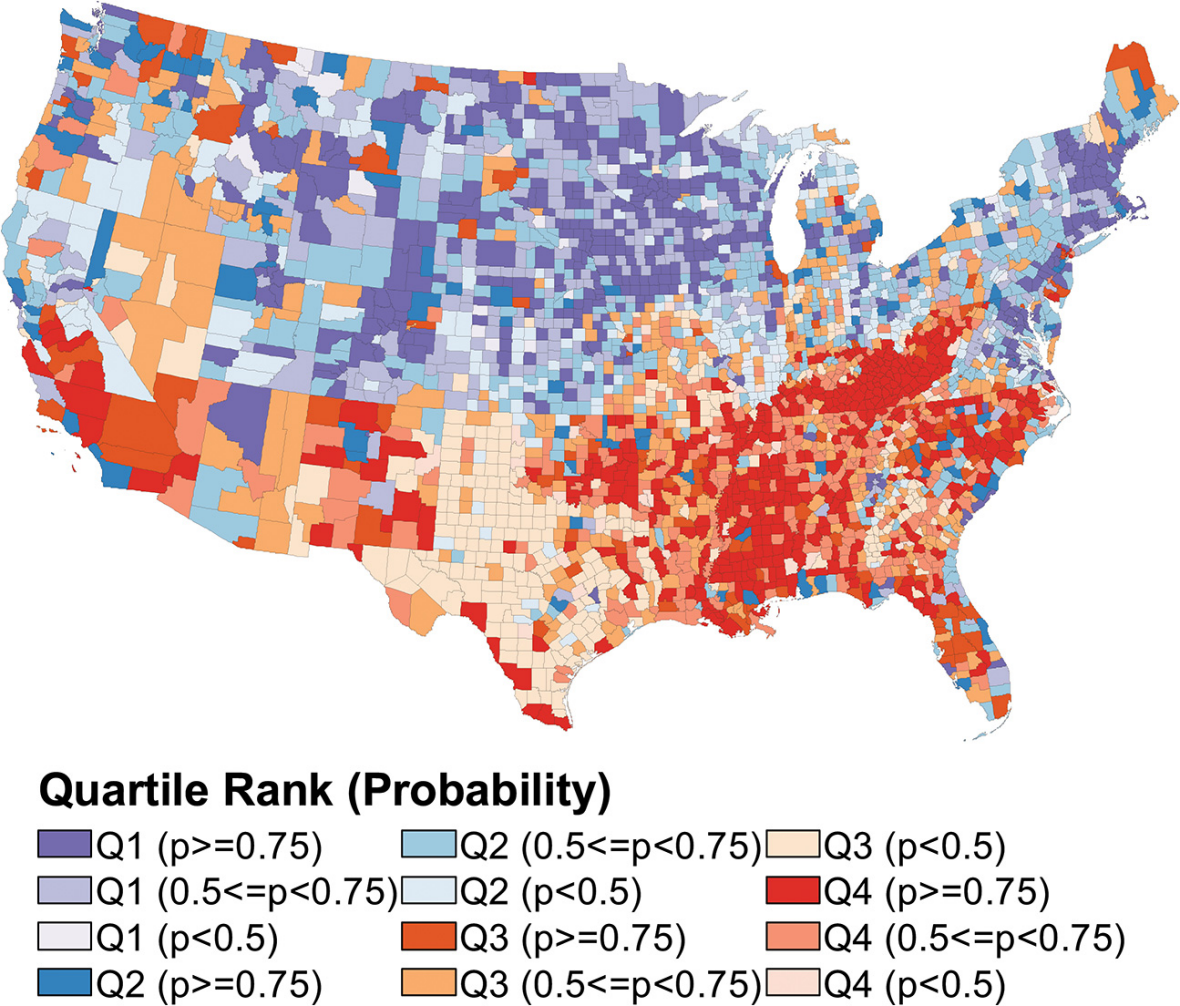
Choropleth Map of U.S. County Rank Performance in Fair or Poor Health Prevalence.

**Fig 4:**
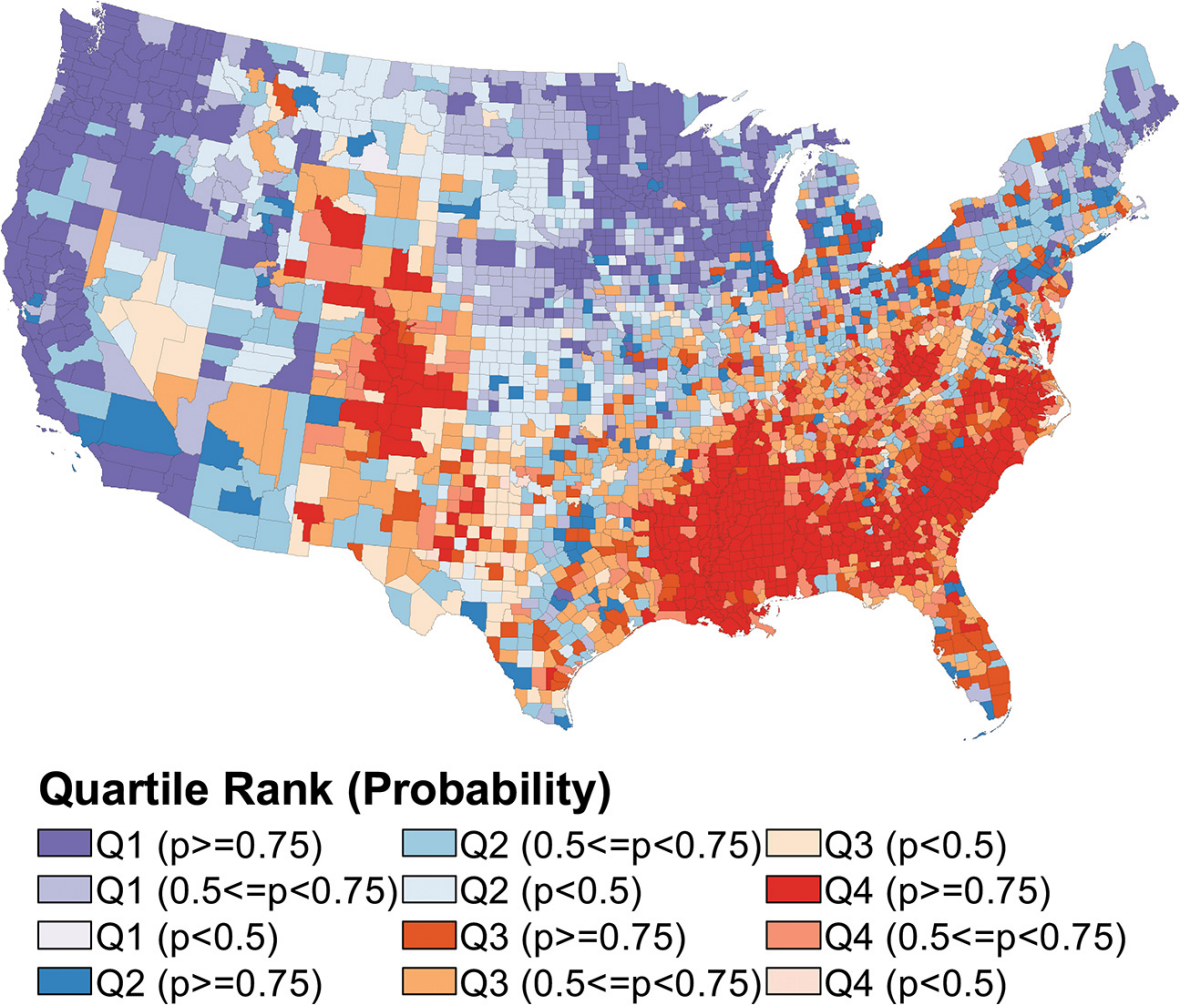
Choropleth Map of U.S. County Rank Performance in Low Birth Weight Births.

Fig 1A–1C show U.S. counties mapped by quartile for premature death (quartile 1 representing the “healthiest” counties, quartile 4 representing the “least healthy”), based on the independent, longitudinal, and joint outcome models. These maps show differences in our ability to distinguish among counties based on our modeling approach. Ideally, a map would have very few light-colored counties, indicating that counties ranked in the same quartile across the posterior samples. The map representing rank performance from the longitudinal model (Fig 1B), though similar to the independent model (Fig 1A), shows greater rank precision. Seventy-one percent of counties rank with high certainty (p ≥ 0.75) in their assigned quartiles using posterior samples from the longitudinal model; using posterior samples from the cross-sectional model, only 59% of counties rank with high certainty (p ≥ 0.75) in a quartile. Conversely, Fig 1C demonstrates how differences among counties are attenuated when using posterior samples from the joint outcome model, with only 47% of counties ranking in their respective quartiles with high certainty (p ≥ 0.75). In these three maps, counties in the Southeastern United States rank among the least healthy, whereas the Midwest, New England, and coastal California rank among the healthiest regions.

Fig 2A and 2B show U.S. counties mapped by quartile and precision for poor mental health days, based on posterior ranks from the independent and joint outcome models. The precision of results from the independent model are poor for this measure, with nearly 20% of counties ranking in their respective quartiles with low certainty (p < 0.5). Only 14% of counties rank with such uncertainty based on posterior samples from the joint outcome model. Poor physical health days do not demonstrate notable improvement with a joint modeling approach, as greater than 20% of counties rank with low certainty in their assigned quartiles in both the univariate and joint outcome models (maps not shown). For both measures, high-performing (healthier) counties tend to cluster in the Plains states and along the mid-Atlantic seaboard. Low-performing counties are grouped in the states of Oklahoma, Kentucky, Mississippi, and Alabama.

In contrast to improvements in rank certainty that result from the longitudinal models for premature death and the joint outcomes model for poor mental health days, rank certainty based on the independent and joint outcome models is similar for percent reporting fair or poor health and percent of births with low birth weight (Figs 3 and 4 show the map results for the univariate models for fair/poor health and low birth weight births, respectively). The geographic distribution of counties ranking in the top and bottom quartiles for fair or poor health mimics that of the premature death measure: New England and the Midwest tend to rank best, with Southeastern counties disproportionately ranking in the lower quartile. High-performing counties in the low birth weight measure are clustered in the Upper Midwest and along the West coast. Counties that rank in the bottom quartile for low birth weight births are commonly located in the Southeast as well as the frontier states of Wyoming and Colorado.

## Discussion

Overall, the expanded models in this paper—using either longitudinal or pooled data—can improve rank estimation and precision compared to cross-sectional or univariate models (for motivations for national rankings versus in-state rankings, see Athens et al. 2013 [4]). In the case of pooled or joint outcome models, advantages are not necessarily observed when the measures modeled jointly are insufficiently related, such as fair or poor health prevalence and percent of births with low birth weight. In this case, however, the quality of the estimates and ranks mimic that of the univariate models, and there is no degradation of rank precision through a joint modeling approach.

The finding that posterior samples for premature deaths from the joint outcome model resulted in less rank precision over the univariate models was not anticipated. One cause of this rank degradation is poor model fit for age groups 1–4, 5–14, and 65–74. Mortality rates among the young age groups are particularly low; a number of counties had zero events over the 2004–06 time period. Though we did not allow for distributions to vary within a joint outcome model, using a negative binomial or zero-inflated Poisson model would better replicate the observed data in these age groups [21]. For the oldest age group, ages 65–74, the joint outcome model overestimated the variance in mortality rates. The independent and longitudinal models estimate almost one-third of the variance for the oldest age group compared to the joint outcome model. Though a death among the 65–74 age group is given little weight in the composite YPLL measure, deaths in this age group comprise about 10% of total years of potential life lost, given the very high mortality rates at these ages. Consequently, the variance from the joint outcome model fit for age 65–74 mortality is reflected in the overall premature mortality estimates.

Refinement in rank estimates was the goal of the expanded models explored in this paper. For select measures, we saw improvement in the certainty with which we could assign counties to a specific quartile. Even so, the quartiles for which the most counties are placed with “high certainty” (p ≥ 0.75) are the top and bottom quartiles (Table 10). Ranks for middle-ranking counties remain more volatile. These results are consistent with Hall and Miller’s examination of rank performance in which a small group of “highly performing” (or, alternately, poorly performing) entities tends to remain fixed in rank [22]. Adding more information confirms the rank location of counties at the extremes, but improving precision for middle-ranking counties is more difficult.

Despite these limitations, these models may be useful in addressing another aspect of data sparseness reflected in the *Rankings*. The Institute currently aggregates up to seven years of data for its measures to improve estimate stability. This approach, however, limits the reactivity of the *Rankings* to changes in community health over time. A potential alternative is to report Bayes estimates and ranks based on data averaged over fewer years. Data from earlier years (or from other, related measures) could be leveraged in the modeling stage to provide reasonable estimates over a shorter time period.

## Conclusion

Using longitudinal or pooled outcome data in Bayesian hierarchical models to create empirical estimates of county event rates and ranks in some cases reduced rank intervals, though some joint models had no impact on rank precision. Further, given the fundamentally different variance in mortality rates across age groups, the joint model for mortality resulted in less precision. These results demonstrate the importance of proper selection of measure combinations for joint modeling to improve rank performance. For models that resulted in improvements, those improvements appeared relatively modest. However, these models can be built upon by including all available data—time series, related outcomes, and perhaps other fixed effects—to maximize the performance of the models for rank estimation.
